# Pasteurization of Milk From an HIV-Infected Woman

**DOI:** 10.1155/IDOG/2006/35482

**Published:** 2006

**Authors:** Sergio Verd

**Affiliations:** Paediatric Clinic, 10 Avenu Alejandro Rossello, 07002 Palma, Spain

Drs Giles and Mijch have provided a timely case of an HIV-infected woman elected to exclusively breastfeed applying milk pasteurization techniques [1]. This is the first report of such a case in a developed country.

We would appreciate the opportunity to discuss the effect of pasteurization in this paper. HIV RNA was detected at <250 copies/mL in the first paired specimens of breast milk, both pre- and postpasteurization. Four months later, the breast milk sample prepasteurization had 60 copies/mL and the sample postpasteurization had 80 copies/mL. If the authors have a proof of a well-done pasteurization, these results challenge our knowledge about HIV inactivation by heat treatment of human milk because this method, including devices that can be used in a home setting, has shown to decrease the infectious titer of cell-free HIV-1 by more than 5 logs [2].

An important problem with regard to HIV-infected patients is compliance with treatment. In fact, in this case, the patient ceased her own antiretroviral medication before this pregnancy. By the way, another explanation for these results is that this patient had neglected the pasteurization of her breast milk, because postpasteurization specimens reported the same or an increase in copy number with respect to prepasteurization specimens.

If this is the case, Giles and Mijch have taught us a lot about how to address the remaining challenge of prevention of mother-to-child transmission of HIV through breastfeeding in developed countries. The way forward to treat these cases must include careful selection of patients to make sure of the compliance with treatment, milk bank pasteurization to strengthen the prohibition of using raw mother's milk, and monthly blood test of the baby to discard any increase in copy number. There is place for physicians to support HIV-infected women willing to breastfeed in rich countries, under strict control.

Sergio Verd
